# Glycosaminoglycan binding to soluble CX3CL1 impacts monocyte migration *in vitro*

**DOI:** 10.3389/fimmu.2026.1747705

**Published:** 2026-02-06

**Authors:** Maria Ennemoser, Paula Peinsipp, Tihana Novak, Clara Matteini, Dorian Tauschmann, Tanja Gerlza, Andreas J. Kungl

**Affiliations:** 1 Institute of Pharmaceutical Sciences, Department for Pharmaceutical Chemistry, Karl-Franzens-University of Graz, Graz, Austria; 2 Antagonis Biotherapeutics GmbH, Graz, Austria

**Keywords:** chemokine, chemokine receptor, fractalkine, glycosaminoglycan, heparan sulfate, monocyte

## Abstract

**Introduction:**

In addition to providing shear-resistant cell-cell adhesion in its natural membrane-bound form, the soluble variant of CX3CL1 (or fractalkine) promotes strong cell migration through signaling via its unique G-protein-coupled receptor CX3CR1 on monocytes, macrophages, T-cells, and NK-cells. To induce cell migration, most chemokines benefit from their interaction with the heterogenous group of glycosaminoglycans (GAGs), which act as coreceptors in chemotactic processes. While the interaction of many chemokines with their GAG counterparts has been investigated in detail, the potential interaction of CX3CL1 and GAGs has not yet received sufficient attention.

**Results:**

Here, we show that the bioactive N-terminal, soluble chemokine domain of CX3CL1 (cdCX3CL1) binds to heparan sulfate (HS) as well as to dermatan sulfate (DS or CS-B), exhibiting Kd-values in the mid nanomolar range. Moreover, the removal of monocyte-surface HS reduced cdCX3CL1-induced cell migration, thereby strongly indicating a potential biological relevance of CX3CL1 binding to GAGs. Interaction studies taking into account the extracellular receptor-peptide of CX3CR1 showed that, in addition to binding CX3CL1, CX3CR1 was found to bind to HS as well. Cross-linking cdCX3CL1 and the CX3CR1 peptide led to a ten-fold higher HS binding affinity compared to the isolated proteins.

**Discussion:**

These findings further strengthen the assumption of an extended interaction network, in which GPCR, chemokine, and GAGs affect each other simultaneously.

## 1 Introduction

The family of chemokines represents a class of small, mostly soluble proteins that includes approximately 50 members (1). Based on the positioning of their specific cysteine residues, the family can be further subdivided into four subgroups: -C, -CC, -CXC, and - CX3C. While the -CC and also the CXC group contain a huge number of representatives, until now, only one chemokine belonging to the -CX3C subgroup has been identified: CX3CL1, fractalkine or, due to its abundance within the central nervous system (CNS), neurotactin (2). Chemokines exert their chemotactic activity by interacting with their G-protein coupled receptors (GPCR) located on the surface of immune cells. Typically, however, a certain promiscuity is observed, i.e. one chemokine interacts with several GPC receptors, and one GPC receptor reacts to several chemokines (3, 4). In addition to GPCRs, chemokines make use of the ubiquitously occurring glycosaminoglycans (GAGs), a strongly negatively charged group of polysaccharides that play a pivotal role in the extracellular matrix (5, 6). GAGs are unbranched polysaccharides, consisting of repeating disaccharide units, with a wide variety of sulfation patterns and degrees - except for HA - which can be broadly subdivided into four groups: Heparan sulfate (HS), Chondroitin/Dermatan sulfate (CS/DS), Keratan sulfate (KS), and Hyaluronic acid (HA) (7). Bound to a core protein via a tetra-saccharide linker and thereby forming the so-called “proteoglycans” (PGs), HS and CS, in particular, play an essential role as co-receptors in chemokine-induced cell migration. PGs immobilize chemokines to allow their further interaction with the receptors in the first place (6, 8, 9), and/or to promote the required oligomerization state of the chemokine’s function (10). To date, the importance of GAG-binding has been demonstrated for many chemokines, and respective GAG-binding sites in the proteins’ structure have been identified (8, 9, 11, 12). In the underlying work, we aim to provide this information for CX3CL1.

**Figure 1:**
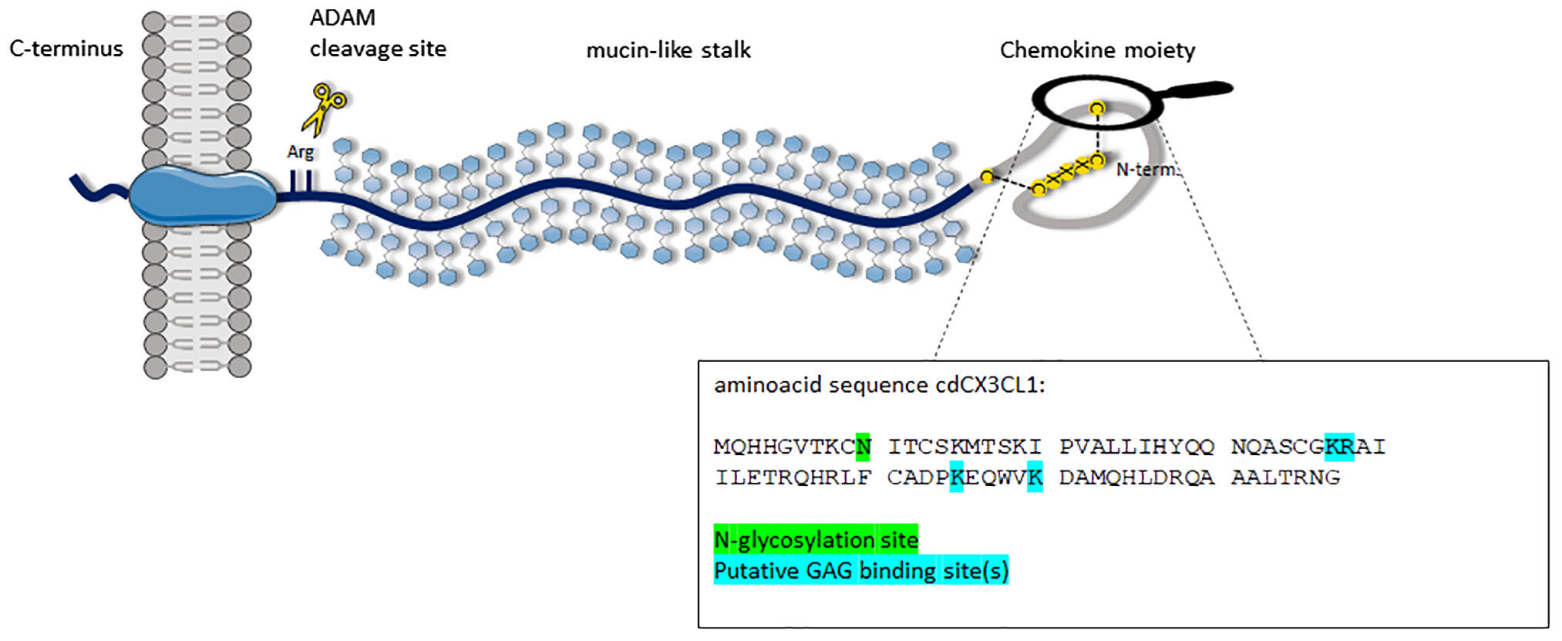
Schematic representation of CX3CL1; from left to right: cytoplasmatic tail, transmembrane domain, highly glycosylated mucin-like stalk and N-terminal chemokine domain; cleavage sites are indicated. In the insert, the amino acid sequence of the chemokine domain of CX3CL1 (cdCX3CL1) is displayed with highlighted GAG-binding site as well as a single N-glycosylation attachment site.

While all chemokines are relatively small and secreted proteins, CX3CL1 is initially expressed as a large transmembrane protein of approximately 40 kDa. As such it consists of a short cytoplasmic tail mainly responsible for oligomerization, a transmembrane α-helical domain assumably for the protein’s oligomerization, a mucin-like stalk and an N-terminal chemokine domain responsible for the interaction with fractalkine’s unique receptor, CX3CR1 (see **Figure 1**) (13, 14). In this membrane-bound form, the chemokine is mainly responsible for the shear-resistant adhesion of CX3CR1-bearing cells. The soluble form of the chemokine consists of the mucin-stalk and the chemokine domain (cdCX3CL1) (15, 16). The soluble form is produced under inflammatory conditions by extensive shedding by two matrix metalloproteinases, namely ADAM 10 and ADAM 17 (17–19).

In contrast to the membrane-bound form, soluble CX3CL1 promotes directed chemotaxis of mainly monocytes and macrophages through the vessel wall towards the inflamed tissue (16). Fractalkine is constitutively expressed, at least in small amounts, almost everywhere on endothelial and epithelial cells in the body, liver, and kidney, but especially within the lungs and on neurons of the CNS (20). Under pathological conditions, pro-inflammatory stimuli by TNFα, IFNγ, IL-1β, and TGF-1 regulate the expression of CX3CL1 and the subsequent release of the soluble chemokine form (21, 22). It acts as a potent chemoattractant for monocytes and macrophages, but also T-cells and NK-cells in a variety of inflammatory and immune diseases (22–27). The specific receptor of CX3CL1, CX3CR1, is expressed on a range of immune cells, mostly monocytes, macrophages, T-cells, NK-cells, and dendritic cells, but also on fibroblasts and microglia (20, 28).

In this way, CX3CL1 is involved in a variety of human diseases (29). In cardiovascular and cerebrovascular diseases, expression of TNFα and IFNγ in phases of high inflammation leads to the overexpression of soluble CX3CL1 and subsequent plaque formation and rupture (29, 30). Rheumatoid arthritis constitutes one of the few diseases for which CX3CL1-targeting therapies have entered clinical testing (31, 32). Recently, great attention has been brought to the involvement of CX3CL1 in a variety of liver and renal disorders. CX3CL1 has been implicated in all forms of glomerular inflammation and endothelial impairment. For example, the chemokine plays a major role in the pathogenesis and progression of diseases such as lupus nephritis, the renal manifestation of systemic lupus erythematosus, and end-stage kidney disease (24, 33–35). In addition, the progressive influence of CX3CL1 in a wide variety of oncological diseases has been demonstrated (29, 36–39), in which CX3CL1 might be of additional value as a potential biomarker.

Although, as mentioned above, the interaction between chemokines and GAGs has been known for a long time and researched extensively ever since (5, 9), very little attention has been paid to the possible interplay between CX3CL1 and this complex family of polysaccharides. Even if occasional interaction- and pull-down studies using heparin, as well as X-ray crystal structures, indicate the possibility of CX3CL1 potentially binding to GAGs (40), clear evidence for the distinct binding of CX3CL1 to HS and the other GAGs is still missing. Also, the necessity of this binding for the biological function of the chemokine has not been investigated to date. More so, it has been labeled as not even necessary (20, 40).

In the underlying study we have therefore investigated the binding of CX3CL1 to glycosaminoglycans (GAGs) and its potential role in the chemokine’s (patho)biology. The primary goal was thereby to quantify the affinity and to assess the selectivity of chemokine-GAG interactions as well as to investigate their impact on leukocyte mobilization and migration. We have focused on the chemokine domain of CX3CL1 (termed cdCX3CL1, see **Figure 1**) since the full-length, membrane bound form of the protein is responsible for leukocyte-endothelial attachment rather than for leukocyte activation. We hypothesized that binding to GAGs on immune cells is crucial for CX3CL1 chemotactic activity. Another important point addressed in the underlying study is the synergistic effect of the corresponding chemokine receptor, CX3CR1, on the chemokine-GAG interaction. This triple complex is suggested to play the driving part in CX3CL1 biology. Based on our findings, novel molecular therapies modulating the chemokine-receptor-GAG interactions could be developed.

## 2 Materials and methods

General laboratory equipment and reagents were purchased from Merck (Darmstadt, Germany) unless stated otherwise. All primers used for mutagenesis were ordered from ThermoFisher (Waltham, MA, USA). Heparan sulfate (Fraction III, with an approximate molecular weight 9 kDa) was acquired from Iduron Laboratories (Alderley Edge, UK), whereas unfractionated dermatan sulfate, as well as hyaluronic acid, were purchased from Celsus Laboratories (Cincinnati, OH, USA). Unfractionated GAGs exhibit typically a molecular weight in the range of 25–40 kDa. Enoxaparin (Lovenox^®^ from Sanofi, Paris, France) was used as low molecular weight heparin (molecular weight ranging between 2–8 kDa).

### 2.1 Recombinant protein expression and purification

The *E.coli* codon-optimized huCX3CL1 chemokine domain gene in a pJ411 expression vector was ordered from ATUM (Newark, CA, USA) and transformed in calcium-competent *E.coli* BL21star(DE3) cells. The resulting colonies were used for overnight culture (ONC) in Luria Bertani medium (LB) (20 g/L containing 50 µg/mL kanamycin) at 37°C and 200 rpm. For protein expression in 8 L LB-medium, a 1:1000 dilution of ONC was used and incubated again at 37°C, 200 rpm. After reaching an optical density (600 nm) of > 0.7, protein expression was induced by adding isopropyl-d-D-thiogalactopyranoside (IPTG) to a final concentration of 0.5 mM. Following a 3-hour growth, cells were harvested at 6000 x g for 10 minutes. The resulting protein pellet was then further processed under non-endotoxin-free conditions, according to Falsone et al. (2013) (41). In brief, after cell lysis, the proteins were purified undergoing a three-step chromatographic process including two liquid chromatography steps (fractogel EMD SO3- and SP-sepharose, both from GE Healthcare, Uppsala, Sweden) and one reversed-phase high-performance liquid chromatography on LiChrosphore 100 RP-18 12 µM (Merck, Darmstadt, Germany). To remove high salt concentrations resulting from the on-column refolding, the protein solutions were dialyzed against 1 x PBS (10 mM NaxHyPO4, 137 mM NaCl, pH 7.4). After concentrating to obtain a final protein concentration of 0.5–5 mg/ml using a centrifugal filter with a three kDa cut-off (Amicon Ultra-15, Merck-Millipore, Burlington, MA, USA), the proteins’ identity was verified using Western Blot analyses. The purity was checked with silver staining according to the protocol of the European Molecular Biology laboratory (EMBL) and/or Coomassie brilliant blue staining.

### 2.2 Structural characterization – guanidine HCl-induced protein unfolding

To examine the conformational state of the proteins, a stepwise unfolding was induced by adding increasing concentrations of guanidine HCl, ranging from 1–6 M to 700 nM protein solutions. After an incubation time of 10 minutes, the solution was measured on the Spectrofluorometer FP-6500 from Jasco (Japan), and spectra were recorded at a temperature of 20°C, a speed of 500 nm/min, an excitation wavelength of 280 nm within a range from 300–400 nm. The stepwise addition of a chaotropic agent leads to conformational changes within the protein’s structure, a conformation-dependent quenching in fluorescence intensity, and a curve shift towards higher wavelengths. Three independent measurements were carried out, and the resulting data were analyzed using Origin 8.0.

### 2.3 Isothermal fluorescence titration

Binding affinities with both interaction partners in solution were carried out using the well-established IFT method, according to Gerlza et al. (42), without modifying either of the two binding partners. The stepwise addition of a ligand causes a dose-dependent quenching of the tryptophan-fluorescence intensity by a binding-induced rearrangement of the proteins. In brief, 700 nM protein solutions were measured on the Spectrofluorometer FP-6500 from Jasco (Japan) at a temperature of 20 °C and a scanning speed of 500 nm/min. After an equilibration time of 30 minutes, the individual GAG stocks at concentrations of 0.1 mM and 0.5 mM were added in 0.5 µl steps, always separated by 1 minute. The same procedure was applied for protein-peptide (PSL, Heidelberg, Germany) titrations, but cdCX3CL1 stock solutions of 0.05 mM and 0.2 mM were used. For titrations with the HS-ligand, pCX3CR1-W (H-DQFPESVTEN FEYDDLAEAC YIGDIVVFGW-OH), including the required tryptophan, was used, while interactions with cdCX3CL1 were measured using pCX3CR1 (H-DQFPESVTEN FEYDDLAEAC YIGDIVVFG-OH). All fluorescence measurements were background-corrected, which means that for every ligand addition step a fluorescence emission spectrum was recorded once with and once without the protein under investigation, and the autofluorescence of the ligand was subtracted from the protein spectrum. Typically, the number of technical replicates was three. The resulting normalized mean changes in fluorescence intensity (-ΔF/F0) were plotted against the respective GAG-concentration, and binding isotherms and Kd-values were analyzed by non-linear regression using Origin 8.0 (Origin Microcal Inc., Northampton, MA, USA) and following equation.


$$F=F_{i}+F_{max}[\frac{K_{d}+[protein]+[ligand]-\sqrt{{\left(K_{d}+[protein]+[ligand]\right)}^{2}-4[protein][ligand]}}{2[protein]}]$$

### 2.4 PBMC cell isolation

Among a whole palette of immune cells, which are able to respond to CX3CL1, monocytes have been described as most suited for chemotactic assays using CX3CL1 as a chemoattractant (43). For monocyte isolation, whole human blood from a healthy donor was diluted 1:2 with HBSS -/- (Gibco), layered over Ficoll plaque Plus (GE Healthcare), and separated by density centrifugation. After removing the plasma layer, the buffy coat containing the monocyte fraction was carefully aspirated and washed 3x with HBSS -/-. Cell counting was conducted, and the total cell number was divided by 6, assuming that monocytes were 1/6 of the isolated fraction. Cells were split and treated with recombinant heparinase III (45 µg/ml; in-house production), respectively, for 30 minutes at 37°C. For masking cell-surface HS-chains, anti-HS-antibody (Amsbio, Wales, UK), to a final concentration of 2.5 µg/ml, was added to the monocytes. For masking CX3CR1, anti-CX3CR1-antibody (Fisher Scientific, Hampton, NH, USA) was used in a final concentration of 2 µg/ml. After checking cell viability and count in the Neubauer hemocytometer, 2 x 10^6^ cells/mL were used for the Boyden chamber chemotaxis assay.

The THP-1 monocytic cell line was purchased from the European collection of cell cultures and grown in RPMI 1640 medium (Sigma) containing 10% fetal calf serum, 2 mM glutamine, 100 units/ml penicillin, and 100 units/ml streptomycin at 37 °C and 5% CO2.

### 2.5 Boyden chamber chemotaxis assay

**Figure 2:**
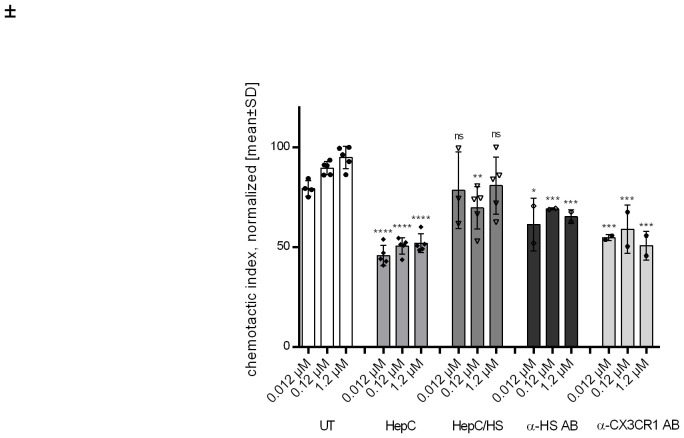
Migrated monocytes upon cdCX3CL1 stimulation for UT (untreated), HepC (heparinase III-treatment), HepC/HS (heparinase III-treatment & HS reconstitution), α-HS AB (anti-HS antibody) and α-CX3CR1 AB (anti-CXCR3 antibody). Conditions were compared to untreated cells for each concentration. Student´s ttest *p < 0.01, **p < 0.01, ***p < 0.001 was considered statistically significant.

**Figure 3:**
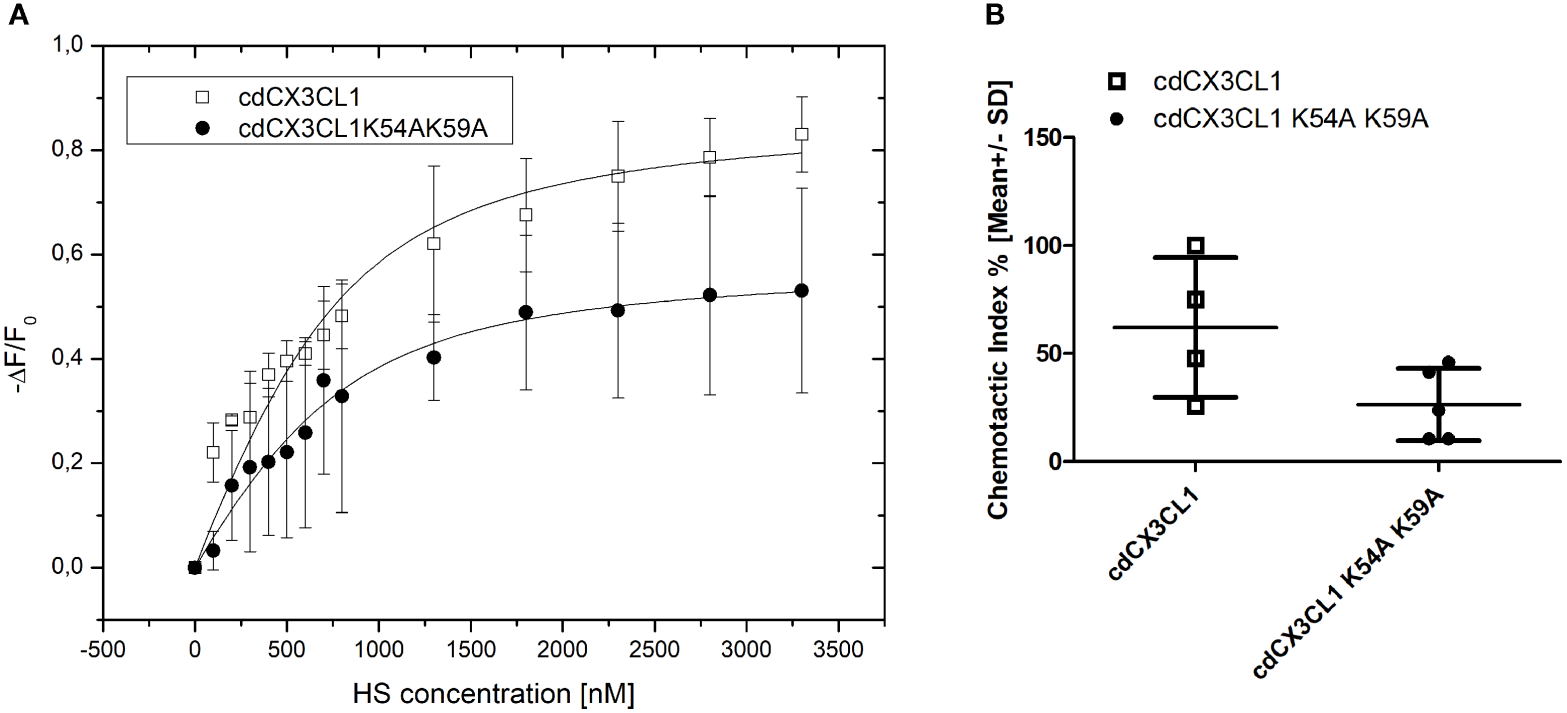
**(A)** Isothermal fluorescence titration data shows lower binding affinity of cdCX3CL1 K54A K59A towards heparan sulfate compared to the binding affinity of wildtype cdCX3CL1 to HS. The depicted data points reflect blank corrected mean ± SD values of three independent measurements; **(B)** cdCX3CL1 and cdCX3CL1 K54A K59A were used as chemoattractant in a Boyden Chamber chemotaxis experiment with THP-1 (model monocytic) cells. cdCX3CL1 K54A K59A decreased the chemotactic index by 57% compared to cdCX3CL1-induced THP-1 chemotaxis (Chemokine concentrations of 1.2 µM were used, chemotactic indices were normalized to 100%; n(cdCX3CL1) = 4; n(cdCX3CL1 K54A K59A) = 5).

To investigate the chemotactic response of monocytes – blood-derived monocytes or THP1 monocytic cell line – to cdCX3CL1 and mutants thereof, a 48-well Boyden chamber (Neuroprobe, Gaithersburg, MD, USA) and a polycarbonate membrane with a pore size of 5 µm (Neuroprobe) were used. Chemokine dilutions ranging from 0.012 – 1.2 µM were placed in the lower compartment and isolated monocytes were placed in the upper compartment to allow directed migration of immune cells through the porous membrane towards a chemokine gradient. Chambers were incubated at 37°C for two hours in 5% CO_2_. Subsequently, the membrane comprising the migrated cells was fixed with Hemocolor fixing solution (Merck) and stained with a Hemacolor staining kit (Merck). Migrated cells were counted under the microscope, and chemotactic indices were calculated, considering the background migration. Technical replicates were typically five (which can be seen in the respective **Figures 2**, **3**). The number of biological replicates was typically two. For data statistics, the unpaired t-test, two-tailed, with 95% confidence interval was applied: * = p<0.1; ** = p<0.01; *** = p<0.001; **** = p<0.0001.

### 2.6 Crosslink experiments

#### 2.6.1 Formaldehyde crosslink

CdCX3CL1 was mixed with varying amounts CX3CR1-receptor peptide and incubated for 30 min at room temperature (RT). Formaldehyde was added to reach a final concentration of 0.75% formaldehyde per sample and incubated for 45 min at RT. To stop the reaction 1.5 M glycine was added to reach a final concentration of 150 mM glycine in each sample. Measuring by densitometry the intensity of the cross-linked complex and the un-complexed partners on SDS-PAGE and silver staining, a 40% cross-link efficacy was estimated.

#### 2.6.2 SDS PAGE and gradient gel for crosslink analysis

15% polyacrylamide and 5% - 20 -% polyacrylamide gradient gels were used for SDS PAGE. The gels ran at 150 V. SDS PAGE gel and sample preparation as well as silver staining was done according to the protocol of the European Molecular Biology laboratory (EMBL).

### 2.7 Size exclusion chromatography

Size-exclusion chromatography was performed on a Hitachi HPLC L-2100 system (Tokyo, Japan) equipped with an autosampler. A Cytiva Superdex 75 (Increase 3.2/300) column was used for separation and 1xPBS (10 mM phosphate buffer and 137 mM NaCl with a pH of 7.35) was used as mobile phase. The flow rate was set to 0.05 ml/min and the temperature in the column oven was set to 25°C. Samples were detected via a diode array detector and analyzed at 280 nm. Prior to the measurement, each sample was diluted with 1xPBS to a final concentration of 4 mg/ml and equilibrated for at least 30 min at 4°C before injection. 10 µl of sample were injected per run. Molecular weight standards: Aprotinin was diluted to 3 mg/ml, Cytochrome C to 2 mg/ml, Carbonic anhydrase to 2 mg/ml and BSA to 5 mg/ml (Merck, Germany). The standard solutions were mixed, 10 µl were loaded onto the column and used as references to calculate the molecular weight of fractalkine by comparing retention times which were determined from three independent runs.

## 3 Results

### 3.1 Recombinant protein expression and purification of cdCX3CL1

For our initial GAG-binding experiments, the N-terminal chemokine domain of CX3CL1 (cdCX3CL1) was successfully expressed in *E.coli* BL21Star(DE3) host cells and purified using a 3-step-purification cascade consisting of cation exchange chromatography and rpHPLC (see **Supplementary Figure S1**, **Supplementary Material**). 15% SDS-PAGE combined with silver staining, Coomassie brilliant blue staining as well as western blotting was used to evaluate and identify degradation products as well as sufficient purity of the recombinant protein. The obtained results showed a purity of cdCX3CL1 of ca. 95% in the absence of any degradation products (see **Supplementary Figure S2**, **Supplementary Material**). Structural and conformational characterization using guanidine HCl-induced fluorescence shifts and unfolding as well as Far-UV-circular dichroism confirmed a structurally intact protein by signaling a properly folded α-helix and β-sheets, as well as unstructured domains, which are, in fact, typical for chemokines (see **Supplementary Figure S3**, **Supplementary Material**). The protein was revealed to be relatively stable and, therefore resistant to guanidine hydrochloride (guaHCl), with an unfolding transition point of 2.6 M guaHCl (see **Table 1**; **Supplementary Figure S4**, **Supplementary Material**). The conserved biologic function of cdCX3CL1 in inducing monocyte migration was confirmed in the Boyden chamber chemotaxis assay, resulting in a highest chemotactic indices at an CX3CL1-concentration of 1.2 µM (see **Figure 2**). The bacterial-derived, non-glycosylated recombinant chemokine domain of CX3CL1, cdCX3CL1, was shown hereby to be structurally stable, exhibiting a typical chemokine fold (consistent with several published chemokine structures), and to be chemotactically highly active. The protein was therefore found to be suitable for the subsequent investigation concerning interactions with different GAG members and their impact on monocyte mobilization and migration.

**Table 1:** Determinants of cdCX3CL1 after purification and quality check.

Determinants of cdCX3CL1	
Molecular weight	8638.99 g/mol
pI	9.56
Estimated purity	ca. 95%
Unfolding transition point (c1/2)	2.6 M

### 3.2 The CX3CL1 chemokine domain binds GAGs in solution

To verify the ability of cdCX3CL1 to bind GAGs in solution, isothermal fluorescence titrations (IFTs) were conducted. In these experiments, increasing concentrations of a GAG-ligand were added stepwise to 700 nM of cdCX3CL1 and respective Kd-values were calculated in the following. Here we probed naturally occurring GAGs, including the predominant variants which cover most of the ECM-PGs, heparan sulfate and dermatan sulfate (CS-B), as well as the non-sulfated hyaluronic acid, were taken into consideration. However, it must be considered that all GAGs used *in vitro* are animal-derived and not identical in structure/sequence to the ones contained within the human ECM and that, particularly *in vivo*, their type and sulfation pattern varies from individual to individual (7).

To monitor conformational changes upon GAG-binding in IFT, the intrinsic tryptophan residue (W57) was used as an environmentally sensitive chromophore. The stepwise addition of the ligand to the protein results in conformational changes in the protein’s structure and a subsequent dose-dependent quenching of the fluorescence intensity. The induced decrease in fluorescence was used to generate binding isotherms with specific slope, stiffness, and saturation, which are further used to calculate Kd-values, which give precise information on the binding affinities of the two interaction partners. The low amounts of protein required and the absence of chemical or genetic modification, while still giving highly sensitive results, are the great advantages of this technique (42).

**Figure 4:**
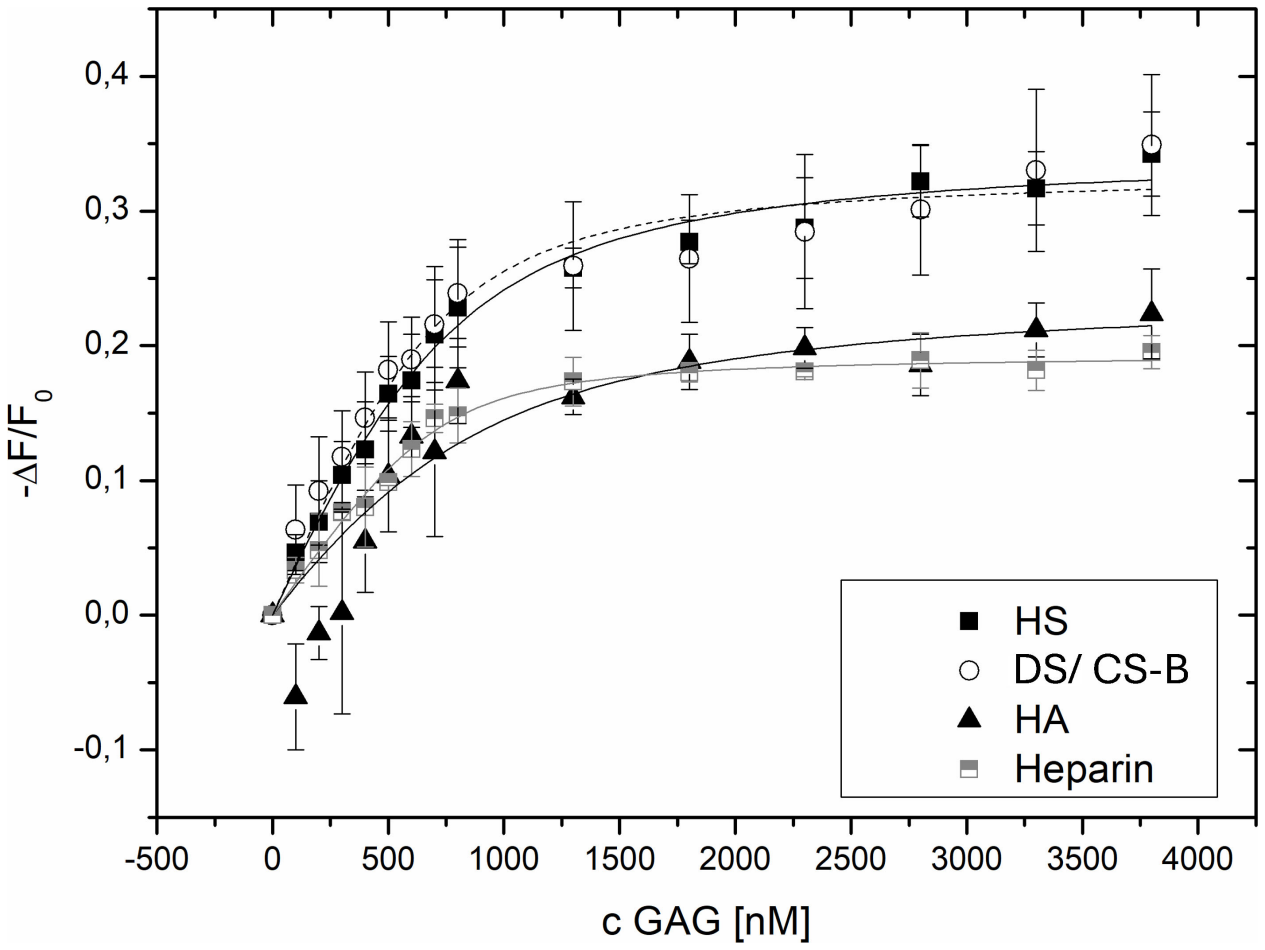
Binding isotherms of cdCX3CL1 to different GAG variants; on the x-axis, increasing ligand addition; on the y-axis, the relative change in fluorescence intensity following ligand addition. The depicted data points reflect blank corrected mean ± SD values of three independent measurements (n=3).

In **Figure 4**, binding isotherms of cdCX3CL1 are depicted, and respective Kd-values of cdCX3CL1 to GAG-ligands are indicated in **Table 2**. Evidently, cdCX3CL1 binds all four GAG-ligands, however, with different affinities and curve shape. The abundantly expressed, and therefore of highest relevance when studying the interactome of the extracellular matrix, HS and DS, show binding isotherms of similar shape and saturation, indicating similar binding properties of cdCX3CL1 to both GAG ligands. These findings are also reflected in similar Kd-values, as depicted in **Table 2**. Interestingly, unexpectedly high affinities were also obtained for the only non-sulfated GAG, HA, referring to the participation of non-charged and/or non-polar amino acids in GAG binding. Remarkably, HA seems to bind cdCX3CL1 in a biphasic manner, pointing to an induced-fit interaction behavior. This means that the chemokine undergoes an initial conformational change at low ligand concentrations due to unspecific ligand interactions, before binding of the HA-ligand continues to a yet changed overall chemokine configuration with a different affinity. This is attributed to multifold unspecific interactions of the carboxyl and N-acetyl groups of HA with cdCX3CL1. The only GAG-ligand, which is of the highest negative charge and not present in the ECM but is, due to their similarities, often used as heparan sulfate surrogate, i.e. heparin, exhibits even stronger binding to cdCX3CL1. These results definitely confirm not only the strong interaction of cdCX3CL1 with GAGs present in the ECM, but also indicate that the N-terminus of the naturally-occurring, full-length membrane-bound chemokine, is engaged in GAG binding and thereby could interfere with physiological and pathophysiological processes.

**Table 2:** IFT-derived Kd values for different GAGs binding to cdCX3CL1.

GAG	Kd ± SD	N
HS	221.5 ± 36.7	5
DS	133.4 ± 42.2	3
HA	384.5 ± 284.9	3
Heparin	8.8 ± 17.8	2

### 3.3 HS impacts CX3CL1 aggregation

**Figure 5:**
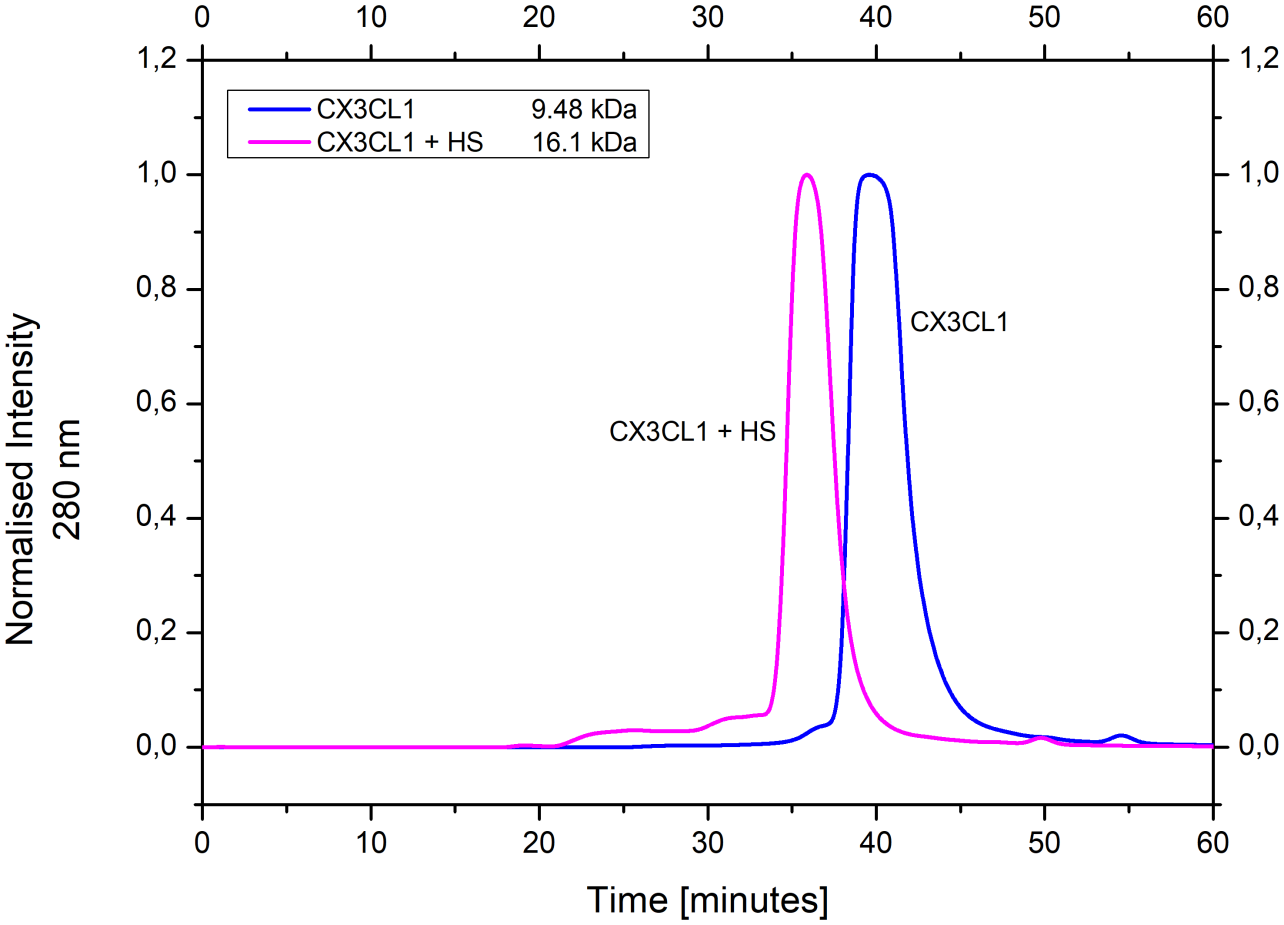
Size exclusion chromatogram of cdCX3CL1 and cdCX3CL1 + HS. The estimated molecular weight of CX3CL1 by this means is 9.48 kDa, while the molecular weight of CX3CL1 + HS is 16.1 kDa (the approximate molecular weight of HS being 9 kDa). Minor peaks indicate the occurrence of larger, HS-induced cdCX3CL1 aggregates.

Size exclusion chromatography was used to determine the molecular size of fractalkine. For this purpose, a mixture of proteins of known molecular weight was separated chromatographically and a calibration curve of the retention times was created. Accordingly, cdCX3CL1 eluted as a 9.48 kDa protein, which is close to the calculated molecular weight of 8.64 kDa, referring to fractalkine as a monomer in solution (**Figure 5**). To determine the effect of HS on fractalkine’s aggregation behavior, cdCX3CL1 was incubated with HS and loaded onto the SEC column under otherwise identical conditions. The CX3CL1-HS major peak eluted ~4 minutes (at 35.85 min) earlier than unliganded CX3CL1 peak maximum (at 39.52 min). In addition, two minor peaks at 25 min and 33 min were detected in the CX3CL1-HS chromatogram (**Figure 5**). The major peak of the CX3CL1-HS trace is indicative of a molecular complex with 16.1 kDa, which is interpreted as the combined molecular weight of the chemokine and the GAG. The two earlier eluting peaks clearly mark the occurrence of higher CX3CL1 aggregates containing HS to various degrees. Since HS may contain more than one binding site for CX3CL1, these aggregates could either represent linearly aligned single chemokine molecules on the GAG ligand, or it could be speculated that HS-binding induces a chemokine conformation which promotes self-aggregation as has been postulated for other chemokines.

### 3.4 Influence of HS-binding on CX3CL1-induced monocyte cell migration

To examine the influence of glycosaminoglycans on CX3CL1-induced monocyte cell migration, an *in vitro* chemotaxis assay was performed. Chemotactic activity of cdCX3CL1 was found for concentrations ranging from 0.012 - 1.2 µM. In a next step, we studied the influence of GAGs on CX3CL1-induced monocyte migration which would be in accordance with our recently published impact of GAGs on the immune cells surface on chemotactic processes *in vitro* (44). For this purpose, isolated monocytes were treated with heparinase III (HepC) to enzymatically remove cell-surface HS. As shown in **Figure 2**, the removal of HS leads to monocytes being less responsive to cdCX3CL1 exposure. Interestingly, the addition of exogeneous HS leads to an almost complete recovery of the chemotactic response of monocytes (see lane 3 in **Figure 2**). This indicates that HS conformationally activates cdCX3CL1, most probably by inducing the chemokine to oligomerize, thereby amplifying the chemotactic activity of the protein. The removal of HS decreased the chemotactic response of monocytes by 50% compared to the cdCX3CL1 untreated cells (a similar effect was observed by applying chondroitinase ABC; data not shown). Masking all HS-chains using an anti-HS-antibody, or masking the GPCR itself by using an anti-CX3CR1 antibody leads to a lesser chemotactic reduction of about 50-60% compared to the untreated monocytes (**Figure 2**). The similar reduction of the chemotactic index following treatment with enzyme and antibodies points in the direction of a similarly accessible amount of cell surface GAGs, which are partly freely amenable and partly bound to the chemokine receptor.

Taken together, these findings evidence once more the presence of proteoglycans on the immune cells surface and the further involvement of these in cell migration. Interestingly, we have seen in our prior publication that CXCL8-mediated neutrophil migration is only slightly affected by CS digestion, whereas CCL2-mediated monocyte chemotaxis is in fact also driven by CS (44). This effect is also visible in CX3CL1-mediated monocyte chemotaxis in this study. As already shown for many other chemokines, also CX3CL1, despite its unique position in the chemokine family as a transmembrane protein, seems to gain migratory benefits from its binding to glycosaminoglycans. However, due to its nature as a constitutive membrane-bound protein but shed form in inflammation, our assays do not clearly indicate whether only the soluble form or both, do use the GAG-axis as a co-receptor for the interaction with CX3CR1.

### 3.5 Identification of the CX3CL1’s GAG-binding site

In order to find out in which region of the cdCX3CL1 molecule the GAG-binding site is located, we have designed, expressed and purified a number of lysine-against-alanine replacement mutants. The design followed a structure-based protocol in which we have identified the putative GAG-binding site of cdCX3CL1 to consist of the amino acids K36, R37, K54 and K59 (see **Supplementary Figure S5**). Out of the combinatorial alanine-replaced mutant proteins K36A/R37A, K54A/K59A, K36A/K54A, and K36A/K54A/K59A, the K54A/K59A variant exhibited the strongest reduction of the heparan sulfate binding affinity to a K_d_ value of 430 nM (see **Figure 3A**; compare to wildtype values in **Table 2**). This was paralleled by an equally strong reduction of the chemotactic index of this mutant compared to the wildtype chemokine at a concentration of 1.2 µM (**Figure 3B**; a THP-1 monocytic cell line was used for screening the chemotactic activity of the CX3CL1 mutants because the number of CX3CR1(+) cells derived from whole human blood was insufficient for the required side-by-side assaying). Although we couldn’t achieve a complete knock-out of GAG-binding and monocyte mobilization/migration, we were able to show where the GAG-binding domain is located and that a reduced GAG-binding affinity leads to a reduced chemotactic activity.

### 3.6 The CX3CL1 interaction network – beyond glycosaminoglycans

Chemokines, glycosaminoglycans, proteoglycans, and their respective receptors form a complex interaction network to fulfill their diverse biological functions within the ECM. Up to now, the specific interaction of chemokines and their GPC receptor(s) and the underlying mechanisms and signaling cascades have already been elucidated. As for CX3CL1, it exclusively binds to its receptor CX3CR1 with affinities in the low nanomolar to the picomolar range (45). Over the course of our work with CX3CL1, we are not only trying to set a focus on the isolated CX3CL1-GAG-axis, but we were also striving to address the whole network around this unique chemokine by additionally considering the chemokine-receptor-co-receptor axis. A potential triple interaction complex, consisting of CX3CL1, its receptor CX3CR1, and specific GAGs, might thereby open the door for a better understanding of the biological role of CX3CL1 but might also be applicable to other chemokines.

The minimal-necessary CX3CR1-domain for functional binding to cdCX3CL1 is the initial N-terminal extracellular domain of the receptor (the peptide sequence is shown in **Supplementary Figure S6**). In the form of a synthesized peptide (pCX3CR1), we performed interaction studies, again using IFT, to investigate, on the one hand, the expected binding to cdCX3CL1, and on the other hand, a potential binding of CX3CR1 to HS. cdCX3CL1 was found to bind to its receptor peptide with an affinity in the nanomolar range (Kd = 353 ± 28 nM). Since only a fragment of CX3CR1 was used for these experiments, the affinity is not as high as would have been expected for the entire receptor protein. Other extracellular loops of CX3CR1 could contribute to CX3CL1 binding as well, thereby strengthening the interaction and raising the respective affinity. Interestingly, submicromolar binding was also detected for the receptor peptide to HS (Kd = 899 ± 103 nM), which suggests that all three biomolecules might share an interaction interface (**Supplementary Figure S6**).

**Figure 6:**
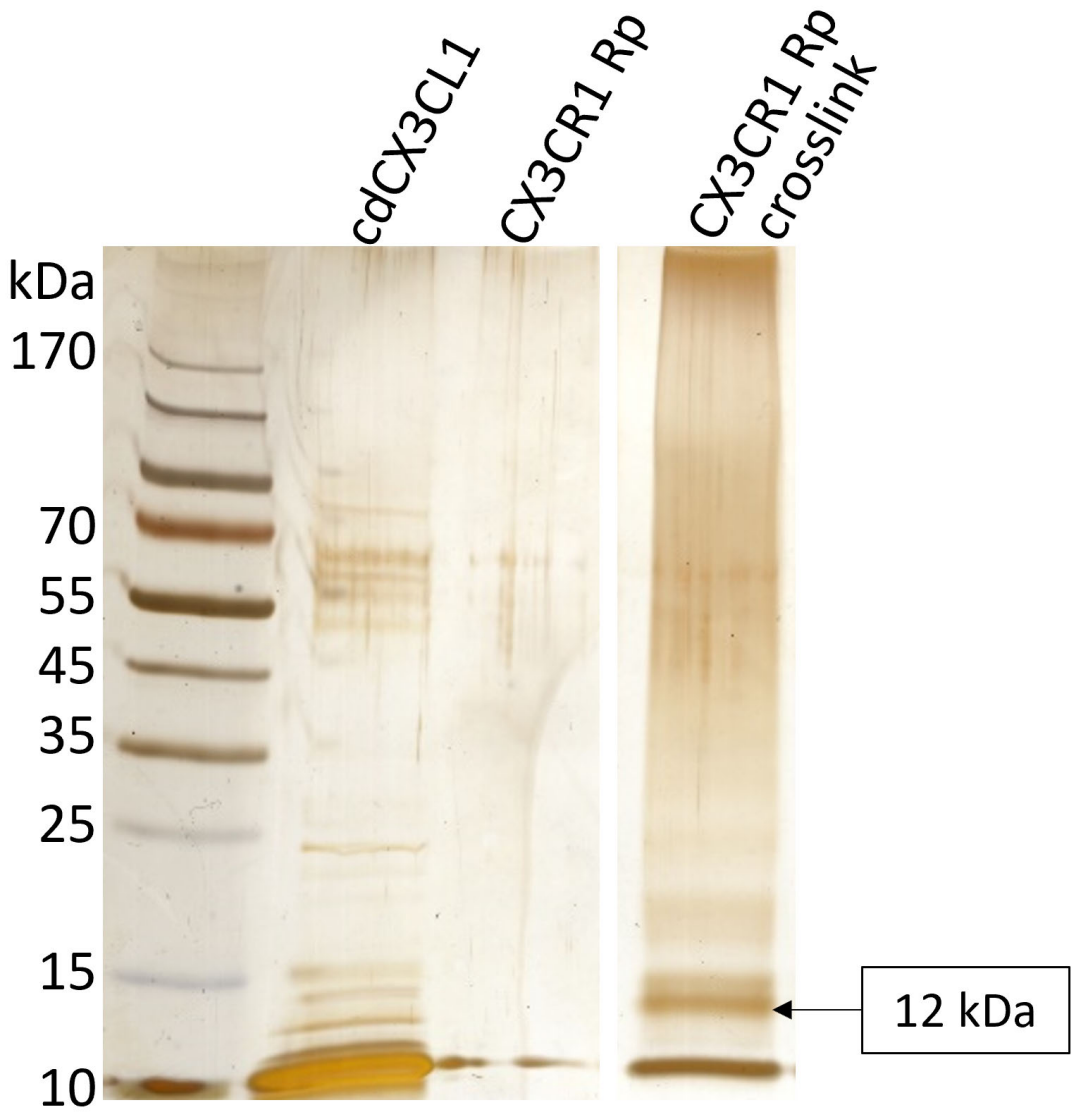
Silver stained SDS gel of cdCX3CL1, CX3CR1 receptor peptide (Rp) and the formaldehyde CX3CL1xCX3CR1 crosslink. The 12 kDa (8639 Da of cdCX3CL1 + 3473 Da CX3CR1 Rp) protein band indicates that the crosslink was successful.

To further investigate the suggested triple complex between CX3CL1, CX3CR1 and HS, crosslink experiments were performed. Formaldehyde was used to form crosslinks between cdCX3CL1 and increasing amounts of the CX3CR1 receptor peptide. After electrophoretic separation by SDS PAGE, a 12 kDa band indicated a 40% successful crosslink, as 12 kDa is the approximate sum of the molecular weights of cdCX3CL1 (calculated Mw = 8639 Da) and the receptor peptide (calculated Mw = 3473 Da). The two samples showing the most intense 12 kDa band (cdCX3CL1 + 4-fold molar excess of CX3CR1-Rp and cdCX3CL1 + 4.32-fold molar excess of CX3CR1-Rp) were pooled and dialyzed with a cutoff of 3500 Da against 1x PBS overnight. The dialysate was investigated by a gradient gel electrophoresis using a 5% - 20% polyacrylamide gel. Again, a clear 12 kDa protein band was visible, which could not be detected in the cdCX3CL1 nor in the CX3CR1-receptor peptide reference sample (**Figure 6**).

**Figure 7:**
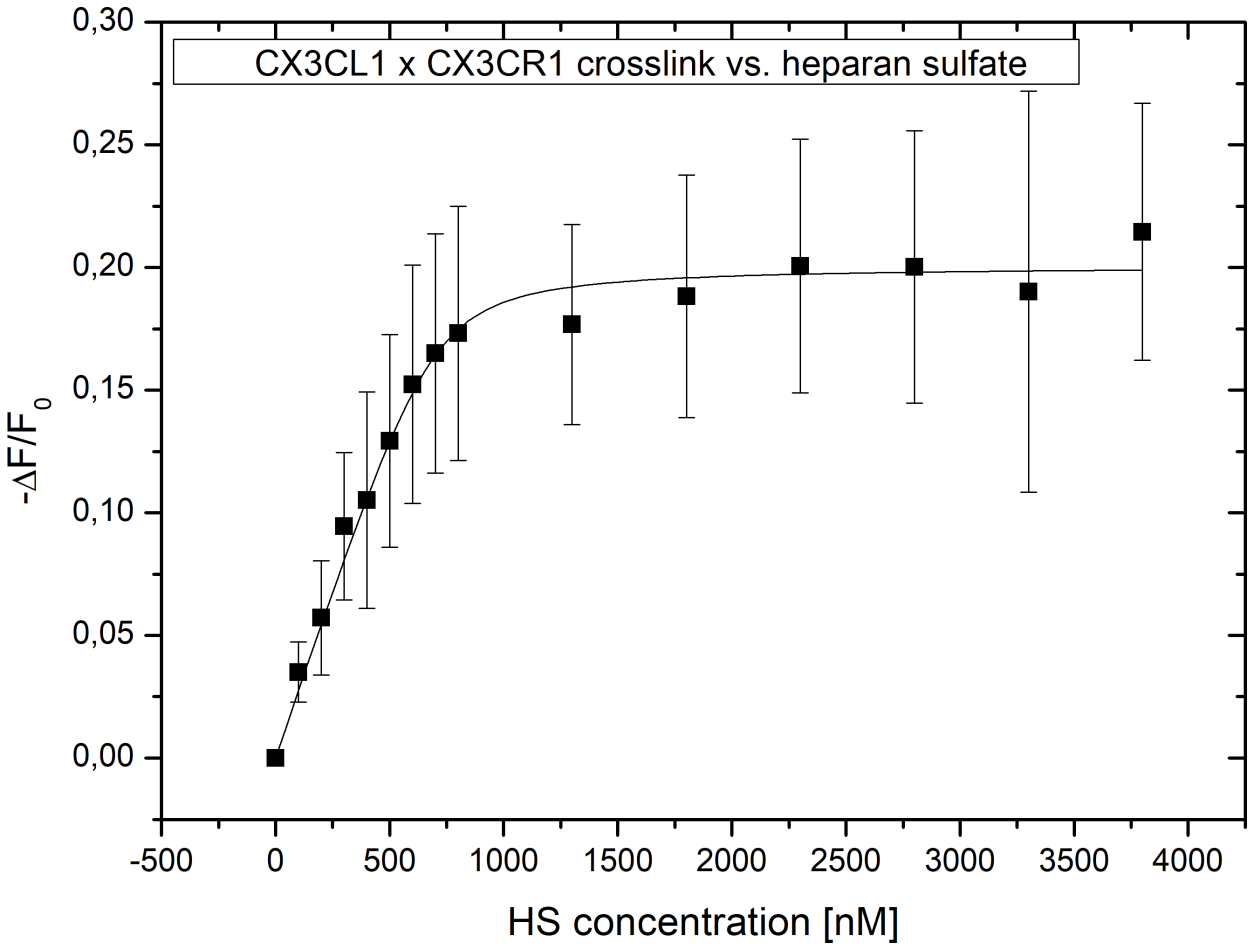
Crosslink of cdCX3CL1 and CX3CR1 Rp binds to HS; binding isotherm of the cdCX3CL1xCX3CR1 crosslinked construct recorded by IFT gave a Kd value of 28 nM. The depicted data points reflect blank corrected mean ± SD values of three independent measurements.

IFT measurements of the dialysate to heparan sulfate (**Figure 7**) additionally revealed that the cross-linked complex of cdCX3CL1 and CX3CR1 has an almost 10-fold higher binding affinity towards HS (Kd = 28 nM) compared to cdCX3CL1 alone (**Table 2**), suggesting that there indeed is a synergistic triple interaction between fractalkine, its receptor and heparan sulfate.

## 4 Discussion

To date, although binding of CX3CL1 to GAGs has already been documented using heparin-sepharose chromatography, X-ray crystal structures of heparin-induced oligomers (42, 46), and in silico models (40, 47), a more detailed investigation is still lacking. Due to the membrane-bound nature of CX3CL1, little biological nor pathobiological significance has been assigned to GAG-binding, which is associated itself with chemokine immobilization. Therefore, an additional association of CX3CL1 to GAGs seemed to be unnecessary. However, as a member of the chemokine protein family, for which GAGs have been identified as co-receptors for many of their functions, it is reasonable to assume that CX3CL1 might interact with this heterogenous group, too. The strong structural similarity of the CX3CL1 chemokine domain (cdCX3CL1) with other chemokines further strengthens this assumption. Here, we have produced the bioactive chemokine domain of CX3CL1 (cdCX3CL1) in *E. coli*. In its natural form, the protein contains one N-glycosylation site (see **Figure 1**, position 10), which remains unmodified when expressed in bacterial cells. Such a modification is typically associated with increased solubility and less with an impact on ligand (receptor or GAG) binding. Therefore, the non-glycosylated form of cdCX3CL1 was considered to be biologically equivalent to the modified form.

We demonstrated the distinct and strong binding of cdCX3CL1 to heparin and the ECM-abundant GAGs HS, DS and HA. The protein’s high affinity to HS and DS in particular, suggests that CX3CL1, like its family members, interacts with the GAG-bearing proteoglycans (PGs) of the ECM – for example the syndecans and the glypicans – to exert its biological functions. Such a strong interaction between biomolecules is expected to affect the protein’s biological and/or pathological function. Previously it was assumed that GAGs located on the endothelium (or in the ECM) of inflamed tissues are required for maximum chemokine-induced immune cell mobilization. Here we have shown, however, that also GAGs localized on monocytes affect cdCX3CL1-induced chemotaxis positively (**Figure 2**). To show this, monocytes were enzymatically treated to remove their cellular HS-chains, or were incubated with an anti-HS antibody: these treatments led to a reduction of cdCX3CL1-induced immune cell migration. This can only be explained by PGs expressed by monocytes and their HS-chains. Previously, we have shown this GAG-dependence also for other immune cells. Their chemokine-induced mobilization and migration potential was found to be dependent upon GAGs co-localized with GPCRs specific for CCL26/eosinophils, CXCL8/neutrophils, and CCL2/monocytes (44, 46–48). It can therefore be concluded that GAGs, co-localized with CX3CR1 on monocytes, are involved in cdCX3CL1-induced migration of these immune cells. These results and conclusions were further corroborated by the newly designed CX3CL1 mutant K54A K59A, which exhibited not only a lower GAG-binding affinity but also a lower chemotactic index in the monocyte Boyden chamber (**Figure 3**). The fact that we couldn’t completely knock-out GAG binding and chemotaxis by these mutations might by due to a non-linear GAG-binding epitope of cdCX3CL1. This would imply that the amino acid region 50–60 of the chemokine (see **Figure 1**) is not the only domain contributing to GAG binding but that another, more distant region is co-responsible for the interaction with the polysaccharide (for example “site I” referred to in Ref. 43, 48). This will be further explored in the future by expanding our cdCX3CL1 mutant library.

Other CX3CR1-positive immune cells, like T-cells and NK cells, are expected to yield similar but not identical results with a different extent of the GAG impact on cell mobilization/migration since each cell type exhibits its characteristic GAG (expression and sulfation) pattern. Consequently, the interplay between GAGs and CX3CR1, when CX3CL1 activates immune cells, is expected to vary dependent upon the cell type. Our results further support our previous findings that HS/DS are generally localized on immune cells where they promote chemokine-induced immune cell mobilization and migration, in addition to endothelially/tissue-expressed GAGs. At this vessel-blood interface, chemokines are known to become immobilized *via* GAGs thereby marking the site of inflammation to which leukocytes are attracted. In this context, we have shown recently by proteomic analysis that following chemokine-GAG binding, the expression pattern of a number of endothelial proteins changed which are involved in cytoskeletal organization, cell adhesion and cell mobility (49).

GAGs are major components of the ECM. In its nature, the ECM acts as a complex functional network, i.e. it is much more than just a space for individual proteins to interact with their distinct counterparts. Therefore, it only makes sense to not only consider the protein-GAG axis individually but to consider it as part of a larger complex. To further explore a potential GAG/chemokine/GPCR triple interaction, protein-protein crosslink experiments were performed. Thereby it was observed, that the crosslinked product of CX3CL1 and its receptor peptide CX3CR1 had an almost 10-fold higher binding affinity towards HS compared to unmodified CX3CL1 (**Figure 7**). Assuming that in full-length CX3CR1, its extracellular loop(s) contribute further to the binding of CX3CL1 and GAGs, the synergistically increased higher affinity towards HS might be even stronger for the full-length protein. Additionally, *via* size exclusion chromatography we were able to show that HS promotes CX3CL1 aggregation. This suggests that HS, having presumably more than one binding site for CX3CL1, leads to an amplification of the chemotactic signal by offering more than one chemokine to its GPCR. We have recently shown, that another ECM component, the protein tenascin-C, modulates the activity of several chemokines. We may therefore conclude, that therapeutically targeting chemokines or their GPC receptors needs to take into account the natural ECM environment of these proteins. This is expected to play a role also for the membrane-bound, full-length CX3CL1. Since this protein form is mainly responsible for leukocyte adhesion to endothelial cells, cell-surface GAGs and GAGs contained in the ECM are expected to play a role in the CX3CL1-mediated cell adhesion through its chemokine domain.

## Data Availability

The original contributions presented in the study are included in the article/**Supplementary Material**. Further inquiries can be directed to the corresponding author.

## Glossary

AIDS: Acquired immunodeficiency syndrome
c-ABC: Chondroitinase ABC
cdCX3CL1: Chemokine domain CX3CL1
CS: Chondroitin sulfate
DS: Dermatan sulfate (Chondroitin sulfate B)
E.coli: Escherichia coli
ECM: Extracellular matrix
ECM-PG: Extracellular matrix- proteoglycan
GAG: Glycosaminoglycan
GPCR: G-protein coupled receptor
HA: Hyaluronic acid
HBSS: Hanks’
HepC: Heparinase III
HPLC: High performance liquid chromatography
HS: Heparan sulfate
ICAM: Intracellular adhesion molecule
IFNγ: Interferon γ
IFT: Isothermal fluorescence titration
IL-1β: Interleukin 1β
IPTG: isopropyl-d-D-thiogalactopyranoside
KS: Keratan sulfate
LB medium: Luria Bertani medium
nCX3CL1: Negative CX3CL1 (receptor knockout)
NK-cells: Natural killer cells
ONC: Overnight culture
PBMC: Peripheral blood mononuclear cells
PBS: Phosphate buffered saline
PCR: Polymerase chain reaction
PG: proteoglycan
pI: Isoelectric point
Rp: Receptor peptide
rpHPLC: Reversed phase high performance liquid chromatography
RT: Room temperature
SDS PAGE: Sodium dodecyl sulfate polyacrylamid gel electrophoresis
SEC: Size exclusion chromatography
TGF-1: Transforming growth factor 1
TNFα: Tumor necrosis factor α
VCAM: Vascular cell adhesion molecule
α-CX3CR1 AB: Anti-CX3CR1 antibody
α-HS AB: Anti- heparan sulfate antibody.
