# Supplementary material for: Glycosaminoglycan binding to soluble CX3CL1 impacts monocyte migration in vitro
Source: Front Immunol. 2026 Feb 6;17:1747705. doi: 10.3389/fimmu.2026.1747705 (PMC12920477; doi:10.3389/fimmu.2026.1747705)
Supplement: Supplementary file 1 [file Table1.docx]

Supplementary Material

## Supplementary Figures


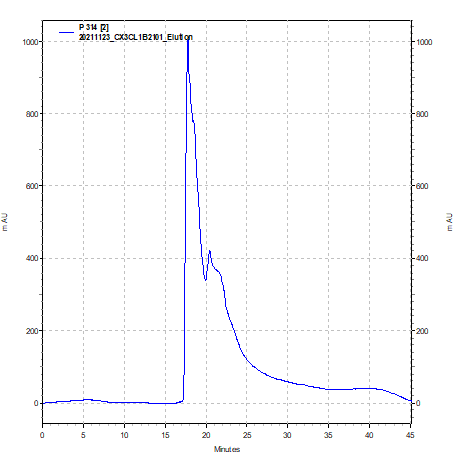

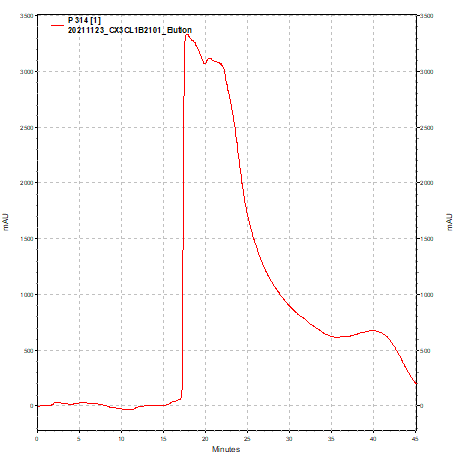


A

C

B

D

**Supplementary Figure S1.** Purification of cdCX3CL1 by FPLC and HPLC. (A) First FPLC purification step using SP-Sepharose column. (B & C) Reversed phase HPLC purification step. Detection was accomplished by absorption at 220 nm (B, red trace) and 285 nm (C, blue trace). (D) Second FPLC purification step using SP-Sepharose column.


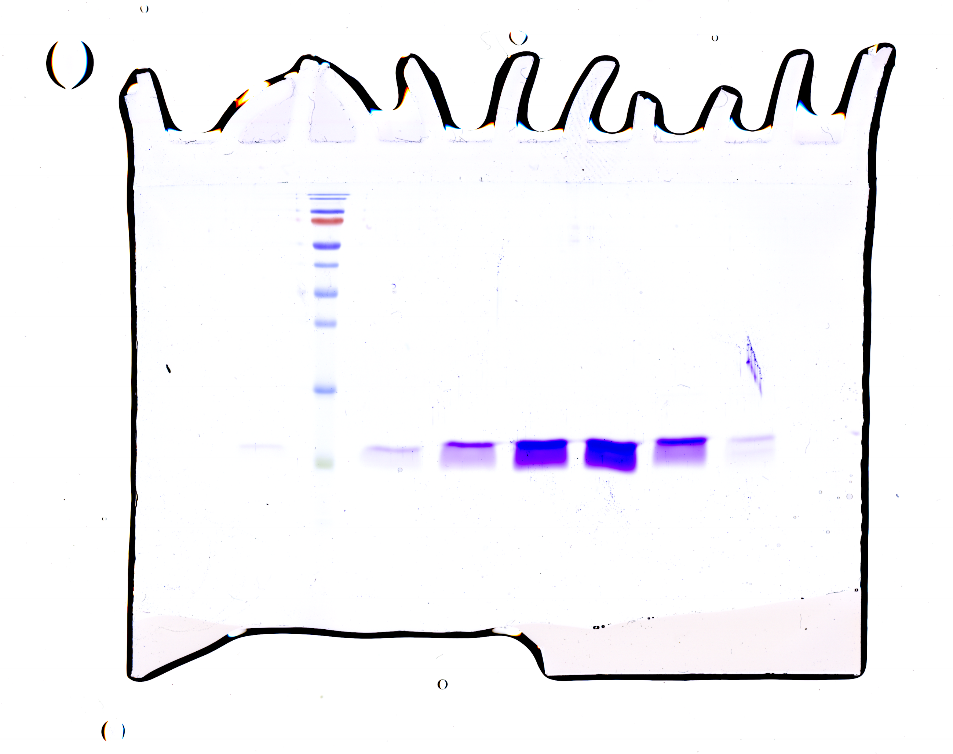


M

increasing
concentration
of cdCX3CL1

Supplementary Figure S2. Purity control of recombinantly expressed cdCX3CL1 by 15% SDS-PAGE. M stands for Molecular weight marker (PAGE Ruler, ThermoFisher Cat#26617)





Supplementary Figure S3: Far-UV spectrum (mean residue ellipticity, MRE) of cdCX3CL1. Secondary structure content was calculated using BestSel.







B

A

Supplementary Figure S4: Chaotrope-induced unfolding of cdCX3CL1. (A) Fluorescence-detected unfolding curve of cdCX3CL1 with unfolding transition point c(1/2) = 2.6 M GuaHCl; (B) fluorescence maximum shift.


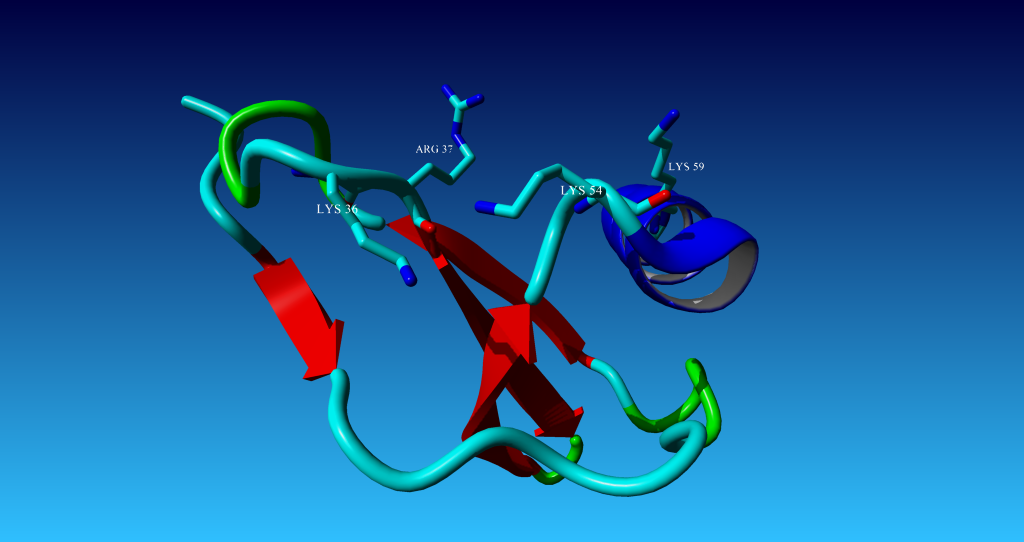


Supplementary Figure S5: Structure of cdCX3CL1 highlightning the potential GAG-binding site of the protein K36, R37, K54 and K59


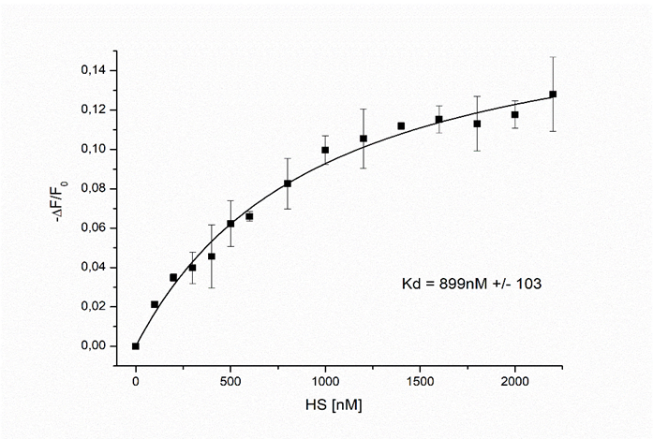

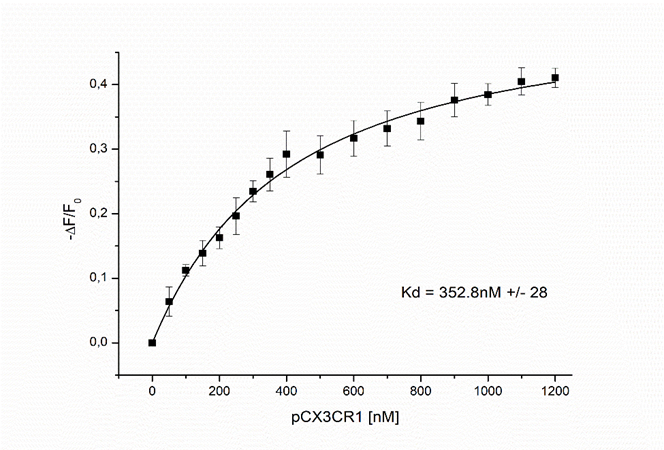


A

B

Supplementary Figure S6: Isothermal fluorescence titration curves of (A) cdCX3CL1 vs the receptor peptide pCX3CR1 and (B) the receptor peptide pCX3CR1 vs HS. The sequence of the receptor peptide is
